# Urbanization and Spatial Aggregation Impair Multifunctionality in Urban Vacant Lots

**DOI:** 10.1002/ece3.72995

**Published:** 2026-01-28

**Authors:** Yuki Iwachido, Himari Katsuhara, Kaho Maehara, Mahoro Tomitaka, Kensuke Seto, Shun Nonaka, Masayuki Ushio, Maiko Kagami, Takehiro Sasaki

**Affiliations:** ^1^ Graduate School of Environment and Information Sciences, Yokohama National University Yokohama Japan; ^2^ Tokyo Metropolitan Research Institute for Environmental Protection Tokyo Japan; ^3^ College of Urban Science, Yokohama National University Yokohama Japan; ^4^ Sugadaira Research Station, Mountain Science Center, University of Tsukuba Ueda Japan; ^5^ Department of Ocean Science The Hong Kong University of Science and Technology Kowloon Hong Kong SAR; ^6^ Institute of Multidisciplinary Sciences, Yokohama National University Yokohama Japan

**Keywords:** bacteria, ecosystem function, fungi, multiple‐taxa, plants, urban shrinkage, urbanization, vacant lots

## Abstract

Urban shrinkage, driven by population decline rather than expansion, is an emerging concern in many developed countries. This demographic shift increases the prevalence of novel green spaces, such as vacant lots, prompting interest in their potential to enhance urban biodiversity and ecosystem multifunctionality. However, biodiversity‐ecosystem multifunctionality relationships in vacant lots remain largely unexamined. We investigated 69 vacant lots in Yokohama, Japan, a city facing potential population decline, by quantifying six environmental factors, five ecosystem functions, and taxonomic and functional diversity and composition of plants, bacteria, and fungi. We used structural equation modelling to analyse the direct and indirect effects of environmental and ecological variables on ecosystem function and multifunctionality. Additionally, to account for trade‐offs and synergies among ecosystem functions, we examined the relationships between environmental factors and multifunctionality using a multiple‐threshold approach and generalized linear mixed models. Our results indicate that environmental factors exerted a dominant influence on ecosystem functions, although all components (environment, plants, and microbes) played a role. Specifically, soil moisture directly enhanced several ecosystem functions and average multifunctionality. In contrast, spatial aggregation of vacant lots indirectly impaired them, mediated by increased plant richness and altered fungal composition. Moreover, urbanization indirectly affected all ecosystem functions and exerted a direct negative effect on average multifunctionality, with its negative effects intensifying at elevated multifunctionality thresholds. These findings highlight that the multifunctionality of urban vacant lots is intricately shaped by environmental factors mediated by diverse taxa. Given that dispersed vacant lot configurations in less urbanized areas may enhance multifunctionality but reduce plant diversity, future urban planning in shrinking cities should balance biodiversity conservation with the enhancement of ecosystem multifunctionality through strategic spatial configuration.

## Graphical abstract

This study provides the first assessment of biodiversity and ecosystem multifunctionality in urban vacant lots. Although urbanization and spatial aggregation of vacant lots impaired multifunctionality, soil moisture improved it.

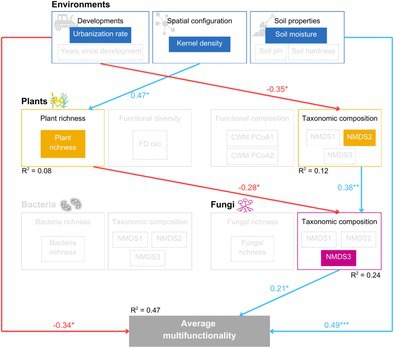

## 1 Introduction

Rapid urbanization adversely impacts global biodiversity (Li et al. 2022; Simkin et al. 2022). However, urban ecosystems, which support diverse flora and fauna, provide essential ecosystem functions and services, such as nutrient cycling and stormwater retention, for over half of the global population (Luederitz et al. 2015; Schwarz et al. 2017; United Nations 2019). Therefore, urban planning has focused on preserving natural areas to mitigate the decline in biodiversity and ecosystem functions caused by the loss of green spaces due to urbanization (Dearborn and Kark 2010). However, many developed countries are currently experiencing urban population declines (United Nations 2019), with approximately 40% of cities in Europe and East Asia projected to shrink (Sun et al. 2024; Zhai et al. 2022). These demographic shifts require a transition from expansion‐focused planning to developing sustainable environments that leverage ecosystem functions in depopulating societies (Marini et al. 2024).

Urban shrinkage generates novel green spaces, including vacant lots, which offer opportunities to enhance biodiversity and ecosystem functions (Johnson et al. 2018; Kelleher et al. 2020; McPhearson et al. 2013; Tsuzuki et al. 2020). In response to pressing urban environmental challenges, such as excessive carbon emissions (IPCC 2023) and urban flooding (Zhang et al. 2018), vacant lots are increasingly recognised for their potential to provide ecosystem functions (Anderson and Minor 2017; Midgley et al. 2021). Although previous research has assessed current ecosystem functions in vacant lots (Kelleher et al. 2020; Kim et al. 2015; McPhearson et al. 2013), the influence of site conditions (e.g., soil characteristics) and the surrounding landscape remains poorly understood. Consequently, the empirical evidence regarding the optimal spatial configuration of vacant lots to maximize their multiple ecosystem functions (multifunctionality) is limited. Urban green space research suggests that land‐sparing, which aggregates green spaces, conserves more species (Soga et al. 2014) and provides higher ecosystem functions (Collas et al. 2017; Stott et al. 2015) than land‐sharing, which disperses them. This implies that the spatial aggregation of vacant lots may also improve biodiversity and ecosystem functions.

Urbanization greatly impacts urban ecosystems (Beninde et al. 2015) and likely influences the ecosystem functions of vacant lots considerably. Urbanization reduces biodiversity and promotes biotic homogenisation through habitat fragmentation and loss (Iwachido et al. 2025; Knop 2016; Marcacci et al. 2021; McKinney 2006). Biodiversity in vacant lots changes over time post‐development (Johnson et al. 2018; Noda et al. 2022), indicating that their ecosystems likely depend on surrounding environments. Therefore, changes in the surrounding environment are expected to influence ecosystem function by altering biodiversity in vacant lots. Specifically, we hypothesize that vacant lots in highly urbanized areas, dominated by stress‐tolerant and highly dispersive species (McKinney 2006), will exhibit lower biodiversity, ecosystem function, and multifunctionality than those in less urbanized areas. Indeed, urban farm studies have indicated that urbanization may adversely impact soil arthropod diversity and indirectly reduce multifunctionality (Schittko et al. 2022).

Ecosystem function is strongly linked to biodiversity, with higher biodiversity generally enhancing multiple ecosystem functions (Chen et al. 2025; Duffy et al. 2017; Tilman et al. 1997). Manipulative plant diversity experiments, in particular, have indicated that increased plant species richness supports greater individual ecosystem functions and multifunctionality (Chen et al. 2025; Hector and Bagchi 2007; Tilman et al. 1997). Furthermore, understanding this relationship requires a multi‐taxa approach, as impacts vary among taxa (Li et al. 2024; Martinez‐Almoyna et al. 2019), and a single‐taxon approach can lead to incomplete assessments of multifunctionality (Moi et al. 2021; Soliveres et al. 2016). Microbial communities (e.g., bacteria, fungi), for instance, are crucial for driving key processes such as nutrient cycling via decomposition processes (Osburn et al. 2023; Singh et al. 2010), making them essential for elucidating biodiversity‐multifunctionality relationships. Indeed, global urban farm studies have demonstrated that soil microbes substantially contribute to multifunctionality (Fan et al. 2023). Therefore, simultaneously considering multiple taxa, including microbes and plants, is crucial for a comprehensive understanding of multifunctionality in urban ecosystems.

**FIGURE 1:**
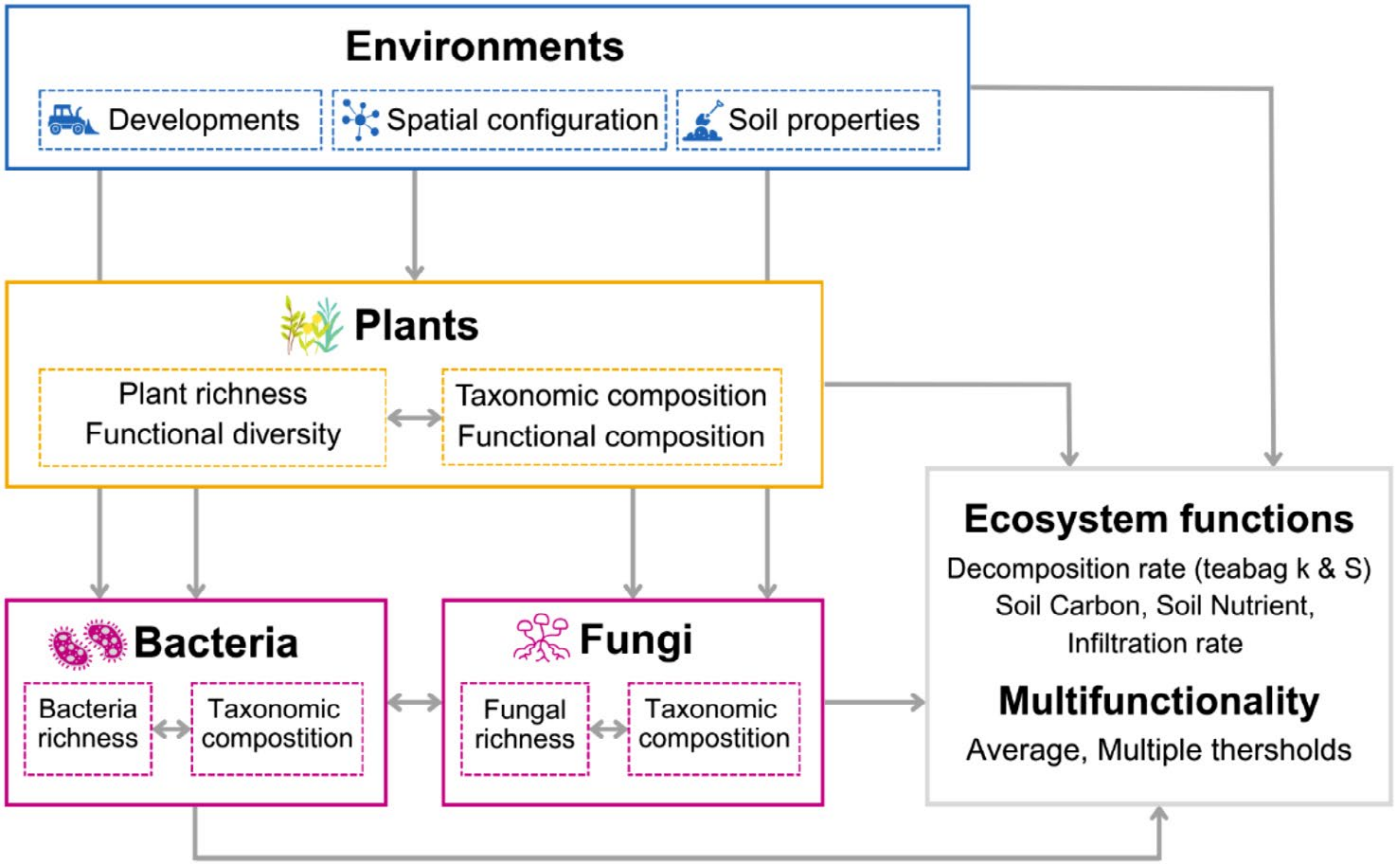
Hypothesised model illustrating environmental change impacts on ecosystem functions and multifunctionality, mediated by shifts in plant and microbial communities. The models were constructed for five ecosystem functions and average multifunctionality, with proposed relationships based on prior studies (details in Figure S1).

In this study, we aimed to assess the effects of environmental and biodiversity components on ecosystem functions and multifunctionality in 69 vacant lots in Yokohama, Japan. The investigated environmental factors included soil pH, soil moisture, soil hardness, urbanization rate, years since development, and vacant lot spatial configuration. The examined biodiversity components included plant, fungal, and bacterial richness and taxonomic composition, and plant functional diversity and composition. To evaluate key ecosystem functions provided by urban vacant lots, we quantified litter decomposition as a proxy for carbon fluxes and nutrient cycling, water infiltration to assess stormwater retention capacity, and soil carbon and nitrogen content as indicators of carbon storage and soil fertility, respectively. We used structural equation modelling (SEM) (Figures 1 and Figure S1) to analyse direct and indirect influences of environmental factors and biodiversity on ecosystem functions and average multifunctionality. Furthermore, we examined the relationship between environmental factors and multifunctionality across varying thresholds using generalized linear mixed models (GLMMs). This study seeks to advance ecological theory and inform urban planning for sustainable urban development by elucidating the mechanisms driving multifunctionality in vacant lots.

## 2 Materials and Methods

### 2.1 Study Area and Sites

The study was conducted across 69 vacant lots in Yokohama, Japan (35°35′–59′ N, 139°49′–65′E). Yokohama, the second‐largest city in Japan, encompasses 438.01 km^2^ with a population of approximately 3.76 million (City of Yokohama 2025). Rapid economic expansion in the 1960s led to urban growth and a decline in green spaces. However, since peaking at 3.78 million in 2021, the population of the city has entered a declining period. Furthermore, it is projected to decrease to approximately 3.5 million by 2050 (City of Yokohama 2024). As a result, a substantial increase in vacant lots and houses is anticipated in the coming decades.

This study focused on 69 vacant lots selected from 250 city‐owned properties with natural vegetative cover (Maehara et al. 2024). These lots, shaped by anthropogenic land modifications (e.g., earth cutting and embankment), have remained undeveloped for approximately 40 years. They are currently secured with fencing to restrict access and are subject to semi‐annual mowing by the city.

### 2.2 Plant Communities and Traits

We established three 5 m transects and two 1 m^2^ plots at the edge of each transect within each study site, totalling 207 transects and 414 plots. Transects were positioned at least 2 m from each other and lot boundaries. We examined plant and microbial communities within each plot.

Plant surveys were conducted in autumn (September–October 2021) and early summer (April–May 2022), visually recording all species present and their respective percent coverages. These data were then pooled at the transect level for subsequent analysis to unify the spatial scales of microbial communities (refer to 2.3). Of the 187 observed plant species, we excluded rare species (those with an occurrence frequency ≤ 5% across transects) and used the remaining 69 species for subsequent analyses.

We focused on eight plant functional traits, leaf height (cm), leaf chlorophyll content (SPAD values), leaf area (cm^2^), leaf dry weight (g), specific leaf area (SLA; cm^2^/g), seed mass (g/1000 seeds), life form (annual, biennial, perennial), and growth form (forbs, graminoids, vines, and shrubs). These traits were selected based on their established links to ecosystem function and urban environments. Leaf traits (SLA, chlorophyll content, leaf area, and dry weight) are linked to nutrient and carbon cycling (Cornelissen et al. 2004; De Deyn et al. 2008). Meanwhile, leaf height, seed mass, life form, and growth form reflect sensitivity to urban environmental conditions. For instance, low leaf height, heavy seeds, annual, or shrub growth forms are thought to facilitate survival and establishment in urban environments (Palma et al. 2017; Williams et al. 2015). For leaf traits, we sampled three fresh leaves of six individuals per species to measure leaf height, leaf chlorophyll content, leaf area, leaf dry weight, and SLA. Detailed methodologies for trait measurements are available by Maehara et al. (2024). Seed mass, life form, and growth form data were compiled from the KEW (Plants of the World Online 2025) and TRY (Kattge et al. 2020) trait databases. Trait data were compiled for all 69 plant species used in the subsequent analyses.

### 2.3 Microbial Communities

Topsoil samples (0–5 cm depth) were collected within each plot in April–May 2022 to characterise microbial communities. Soil samples from two plots per transect were then composited by transect. Microbial diversity was estimated using operational taxonomic units (OTUs) as a proxy for species‐level diversity. To avoid overestimating diversity due to sequencing errors, we first excluded OTUs with a relative abundance below 0.1% (Bokulich et al. 2013). Subsequently, data were rarefied using the *rrarefy* function in the vegan package (Oksanen et al. 2020) to standardise sampling effort across samples. Separate bacterial and fungal community matrices were generated per transect. Detailed methodologies for DNA extraction, library preparation, and sequencing are provided in Maehara et al. (2024).

### 2.4 Environmental Factors

We examined several environmental factors: soil properties, years since development, surrounding land use, and vacant lot spatial configuration. Soil hardness was measured at each plot using a Yamanaka‐type tester, while soil pH (pH meter: D‐71LAB, HORIBA Ltd., Japan) and soil moisture content (mass loss after oven‐drying at 60°C for 24 h) were determined from soil samples collected for microbial analysis. Years since development were derived from historical aerial photographs (1945–2019; GSI map, https://maps.gsi.go.jp/) by subtracting the identified development year from the survey year (2022). The proportion of surrounding land use (urban, farmland, and green spaces) within a 500‐m radius of each site, following previous studies on vacant lots (Blouin et al. 2019; Tsuzuki et al. 2020), was calculated using ArcGIS 10.8 and the High‐Resolution Land Use and Land Cover Map (resolution: 10 m, 2018–2020, JAXA). The spatial configuration of the initial 250 vacant lots was quantified using kernel density estimation via the density.ppp function in the *spatstat.explore* package (Baddeley et al. 2025). A Gaussian kernel was used, with the bandwidth automatically selected using Diggle's method (Diggle 1985). Higher kernel density values indicate a higher degree of spatial aggregation (clustering) of vacant lots, whereas lower values represent a more dispersed spatial pattern.

### 2.5 Ecosystem Functions

We quantified five ecosystem functions metrics relevant to urban vacant lots (Haase et al. 2014; Midgley et al. 2021): total soil carbon (Soil C), total soil nitrogen (Soil N), water infiltration rate, and two litter decomposition parameters (initial decomposition rate: teabag index k, and stabilization factor: teabag index S). Soil C, Soil N, and decomposition parameters are related to carbon and nutrient cycles, while water infiltration rate serves as a proxy for stormwater flood control. Soil C and N contents were measured using a CHNS organic elemental analyzer (UNICUBE, Elementar, Germany) on soil samples. Water infiltration rate (unsaturated hydraulic conductivity) was measured for each transect using a Mini Disk Infiltrometer (METER, CA, USA), calculated according to Zhang (1997). Litter decomposition was assessed by using the standardized tea bags index method (Keuskamp et al. 2013). Tea bags (Lipton Green Tea and Rooibos) were buried at a depth of 8 cm for 3 months (April–July 2022). Following retrieval, drying (65°C for 72 h) and cleaning of plant roots and soil, the remaining mass was used to calculate teabag *k* and *S*.

### 2.6 Statistical Analysis

We quantified the plant, bacterial, and fungal richness and taxonomic composition at each transect. Richness was defined as the number of species for plants and the number of OTUs for bacteria and fungi. Taxonomic composition was assessed through non‐metric multidimensional scaling (NMDS) based on Bray–Curtis dissimilarity in three dimensions (set. seed = 123), implemented via the *metaMDS* function in the vegan package (Oksanen et al. 2020) (Figure S4). For plants, functional diversity was estimated using Rao's quadratic entropy, while functional composition was assessed using community‐weighted mean trait values. Because of correlations among traits, principal coordinate analysis (PCoA) was applied using the *cmdscale* function in the vegan package, with the first and second axes explaining 34.8% and 18.2% of the variation, respectively (Figure S2) (Oksanen et al. 2020). PCoA axis 1 correlated positively with SLA and negatively with seed mass and LA, whereas PCoA axis 2 correlated positively with chlorophyll content and negatively with leaf height. CWM scores for these PCoA axes were calculated and weighted by relative cover.

We quantified individual ecosystem functions and multifunctionality (using both average and multiple‐threshold approaches) at each transect. Each ecosystem function was normalized to a 0–1 scale for comparability. The teabag index k was excluded from multifunctionality dowing to data limitations (*n* = 34; teabag damage and marker loss during mowing). Average multifunctionality was calculated as the average of standardized four ecosystem function metrics. To account for synergies and trade‐offs between ecosystem functions and avoid the arbitrariness of selecting a single threshold, we used the multiple threshold multifunctionality (Byrnes et al. 2014). This allowed us to determine whether the effects of environmental factors on multifunctionality were consistent across low, medium, and high performance levels (Byrnes et al. 2014). Multifunctionality was calculated as the number of ecosystem functions exceeding a given threshold at each transect. These thresholds were defined as a proportion of the maximum observed value for each ecosystem function, ranging from 1% to 99% in 1% increments. Following variable calculations, only 51 transects contained complete datasets owing to teabag loss (resulting from mowing) and limited microbial data availability.

We applied piecewise structural equation modelling (piecewise SEM package; Lefcheck 2016) to examine relationships among biotic variables (i.e., richness, functional diversity, and taxonomic/functional composition), environmental factors (i.e., surrounding land use, years since development, soil characteristics, and spatial configuration of vacant lots), and ecosystem functions (i.e., individual ecosystem functions and average multifunctionality) (Figure 1, Figure S1). First, we created full SEM models based on a priori ecological knowledge (Figure S1) while accounting for correlation errors within plant and microbe datasets. In the SEMs, we modelled richness using GLMMs with a Poisson distribution and the other variables using linear mixed models (LMMs), with site included as a random intercept in all models. After excluding multicollinear predictors (Pearson's *r* > 0.6; e.g., plant/bacteria NMDS1, soil pH; Figure S3), we simplified the models by removing explanatory variables based on the Akaike Information Criterion (AIC) and Fisher's C statistic, achieving an adequate fit (Fisher's C, *p* > 0.05; Table S2). Standardized total effects (STEs) were calculated by summing direct and indirect effects using standardized coefficients of significant predictors (*p* < 0.05).

We used GLMMs with a Poisson distribution to examine how variables identified in the SEM (urbanisation rate, soil moisture, and NMDS axis 3 of fungi) influence multifunctionality across varying thresholds. However, our analysis was limited to thresholds up to 70% because of the limited transects (*n* = 4) exhibiting ecosystem functions exceeding this level. As the indirect effects of urbanization rate and kernel density of vacant lots on average multifunctionality were minimal (STE = 0.03), these variables were excluded from the model. Specifically, multiple‐threshold multifunctionality (the number of functions exceeding a given threshold) was selected as the response variable, urbanization rate, soil moisture, and NMDS axis 3 for fungi were selected as explanatory variables, and site identity was selected as a random intercept. Separate models were constructed for each threshold, and standardized coefficients and 95% confidence intervals for each explanatory variable were estimated.

All statistical analyses were performed in R (version 4.2.2) using the lmerTest (Kuznetsova et al. 2020) and FD (Laliberté et al. 2023) packages.

## 3 Results

**FIGURE 2:**
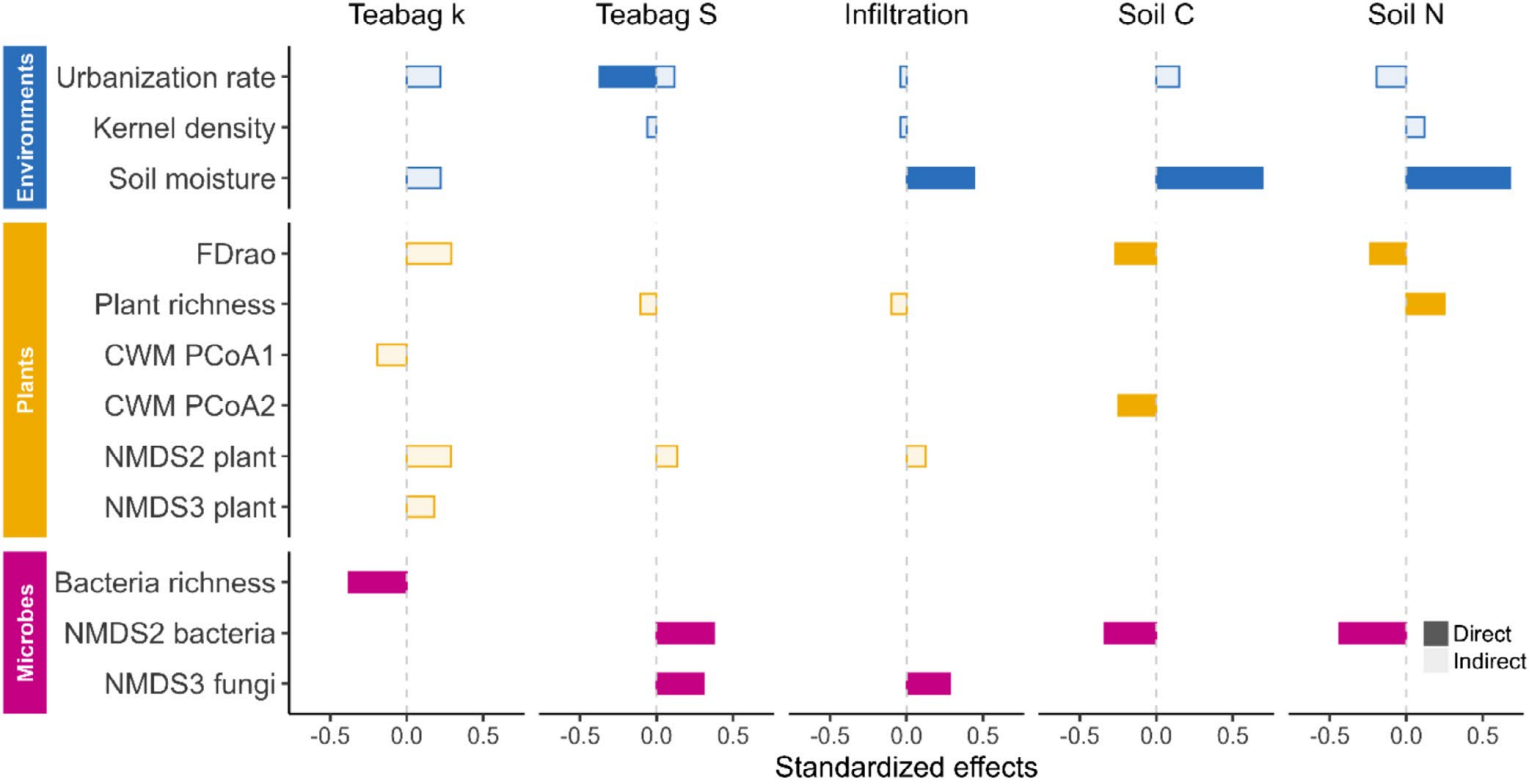
Standardized total effects on each ecosystem function were calculated from best structural equation models. Environmental (urbanization rate, kernel density, and soil moisture), plant (functional diversity [FDrao], plant richness, functional composition [CWM PCoA1 and PCoA2], taxonomic composition [NMDS2 and NMDS3 plant]), and microbial (bacterial richness [number of OTU], bacterial community composition [NMDS2 bacteria], and fungal community composition [NMDS3 fungi]) components significantly influenced ecosystem functions (*p* < 0.05). More details and path coefficients are available in Table S1 and Figures S5–S9.

All ecosystem functions were directly and indirectly influenced by environmental, plant, and microbial components (Figure 2, Table S1). Urbanization rate significantly affected all ecosystem functions (Figure 2), while microbial communities played a key role in shaping these functions (Figure 2). Bacterial richness negatively affected teabag *k* (STE = −0.38; Figure S5). NMDS2 bacteria positively affected teabag *S* (STE = 0.37) but negatively impacted soil C (STE = −0.33) and N (STE = −0.44) (Figures S6, S8, S9). NMDS3 fungi (positively associated with Basidiomycota) positively affected teabag *S* (STE = 0.31) and the infiltration rate (STE = 0.28) (Figures S6 and S7). Soil moisture positively affected infiltration rate (STE = 0.44), soil C (STE = 0.69), and soil N (STE = 0.68). Kernel density had negative indirect effects on teabag *S* (STE = −0.06) and infiltration rate (STE = −0.04) and a positive indirect effect on soil N (STE = 0.12). Plant functional diversity (FDrao) negatively impacted soil C (STE = −0.27) and N (STE = −0.24), while plant richness positively affected soil N (STE = 0.25). Furthermore, plant functional composition (CWM PCoA2) negatively impacted soil C (STE = −0.25).

**FIGURE 3:**
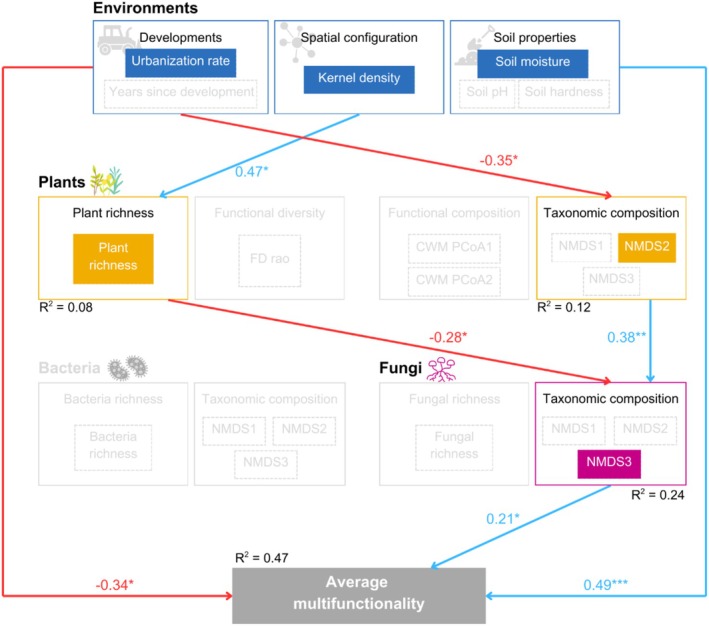
Optimal structural equation model for average multifunctionality drivers (Fisher's C = 23.347, *p*‐value = 0.613, and AIC = 294). Blue and red lines indicate significant positive and negative effects, respectively. Standardized effect sizes are displayed above each path, and statistical significance is indicated by asterisks denoting *p*‐values (* *p* < 0.05, **p < 0.01, ***p < 0.001). The variance explained (*R* ^2^) is shown below the corresponding box. Light grey variables indicate non‐significant effects on average multifunctionality.

**FIGURE 4:**
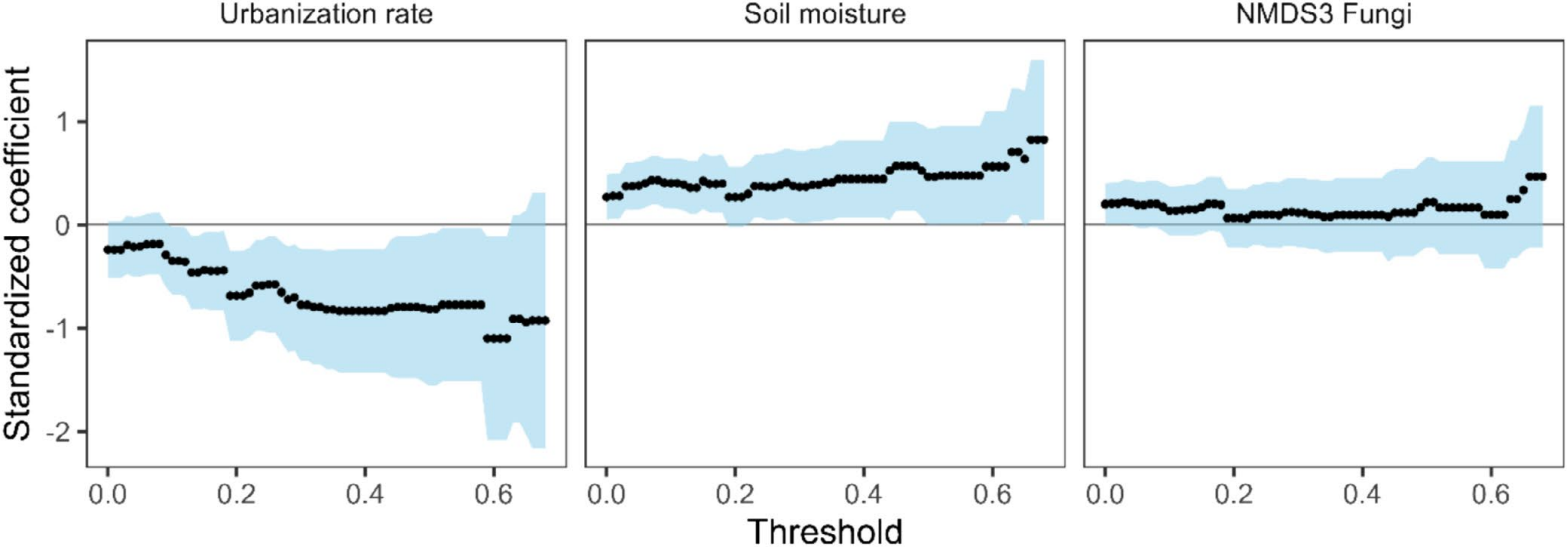
Standardized coefficients, derived from generalized linear mixed‐effects models, illustrate the effects of urbanization rate (left panel), soil moisture (center panel), and NMDS axis 3 for fungi (right panel) on multifunctionality at various thresholds. The points represent the standardized coefficients, while the light blue areas denote the 95% confidence intervals. Significant effects at each threshold are identified via 95% confidence intervals that do not overlap with zero.

Environmental variables primarily influenced average multifunctionality (Figure 3). Soil moisture exhibited a positive effect (STE = 0.49), whereas the urbanization rate demonstrated both negative direct (STE = −0.34) and indirect influences on average multifunctionality, mediated through NMDS2 plant (positively correlated with the relative abundance of *Trifolium pratense* ) and NMDS3 fungi (STE = −0.03 for each pathway). Kernel density exerted a negative indirect effect on average multifunctionality (STE = −0.03) via a pathway that included plant richness and NMDS3 fungi. The multiple‐threshold approach demonstrated a consistent negative impact of the urbanization rate on multifunctionality, which intensified with higher thresholds (Figure 4). Conversely, the positive effect of soil moisture marginally increased at higher thresholds. However, NMDS3 fungi did not significantly affect multifunctionality when evaluated through the multiple‐threshold approach.

## 4 Discussion

This study revealed that plant and microbial components directly and indirectly influenced most ecosystem functions, consistent with previous research emphasising the importance of multiple taxa in ecosystem functioning (Moi et al. 2021; Soliveres et al. 2016). However, the primary drivers varied across different ecosystem functions. Microbial communities strongly affected the initial decomposition rate (teabag *k*) and the stabilization factor (teabag *S*), consistent with their known role in organic matter decomposition (Raza et al. 2023). Bacterial richness was negatively correlated with the initial decomposition rate (teabag *k*), contrasting with previous findings (Maron et al. 2018; Nielsen et al. 2011), likely due to decomposition being driven by specific bacterial taxa rather than overall richness (Li et al. 2025). Stabilization factor (teabag *S*) was positively correlated with the relative abundance of Proteobacteria (positively correlated with NMDS2 bacteria), indicating their role in stabilization. Proteobacteria exhibit high metabolic diversity, including amino acid and protein decomposition (Liu et al. 2024), contributing significantly to decomposition processes. Their dominance in early‐stage coal mine regeneration (Jia et al. 2022) suggests a similar role in decomposition within relatively young green spaces, such as vacant lots. Therefore, specific bacterial phyla (e.g., Proteobacteria) are key for decomposition in vacant lots. Both the stabilization factor (teabag *S*) and the infiltration rate correlated positively with the relative abundance of Basidiomycota (positively correlated with NMDS3 fungi). Basidiomycota likely enhance infiltration by improving soil aggregation because their hyphae bind soil particles (Leifheit et al. 2014) and contribute to litter decomposition as cellulose decomposers (Leifheit et al. 2024; Treseder and Lennon 2015).

Soil C and N were primarily influenced by plant components. Specifically, plant functional diversity negatively affected soil C and N, whereas plant richness had a significant positive effects on soil N and a marginally significant positive effect on soil C (*p* = 0.08). This suggests that increased species with similar traits within the community may enhance soil C and N accumulation through enhanced photosynthetic activity. Soil C was negatively correlated with CWM PCoA2 (where negative values represent high leaf height and high SLA), indicating that dominance of species with greater height and SLA is associated with higher soil C content. These species, characterised by rapid growth and strong competitive ability, may outcompete others for biomass (Bennett et al. 2016; Keddy et al. 2002), ultimately increasing soil C and N.

Soil moisture positively influenced the infiltration rate, soil C and N, and average multifunctionality, likely mediated by increased aboveground and belowground biomass. Meta‐analyses have demonstrated that increased macropores and root channels associated with higher belowground biomass contribute to higher infiltration rates (Shi et al. 2021). Furthermore, increased nutrient supply from plants to soil, driven by greater aboveground biomass, enhances soil C and N contents (Berdugo et al. 2020; Song et al. 2019). Generally, higher soil moisture supports greater biomass and microbial activity, enhancing multifunctionality compared to drier areas (Hu et al. 2021). This positive effect was consistent across all thresholds, indicating that preserving high‐moisture vacant lots benefits multifunctionality regardless of performance levels. Therefore, soil moisture in vacant lots may serve as a key multifunctionality indicator, guiding urban planning to prioritise the conservation of high‐soil moisture vacant lots, while repurposing low‐moisture areas for other uses (e.g., as urban farms and parks). However, although plant biomass is a crucial factor of many ecosystem functions (Cardinale et al. 2012), we were unable to quantify it in this study. Thus, future studies should explicitly examine the relationship between plant biomass and environmental variables in vacant lots.

Kernel density indirectly and positively affected soil N by increasing plant richness, suggesting that spatial aggregation of vacant lots enhances both plant richness and soil nitrogen accumulation. Higher connectivity may facilitate colonisation, explaining increased plant richness at higher kernel density (Brudvig et al. 2009). However, kernel density indirectly negatively affected the stabilization factor (teabag *S*), infiltration rate, and average multifunctionality. While spatial aggregation of vacant lots enhances plant richness, it can simultaneously reduce certain ecosystem functions. These negative effects were mediated through increased plant richness and the relative abundance of Mucoromycota (negatively correlated with NMDS3 fungi). Mucoromycota, including arbuscular mycorrhizal fungi (AMF), are associated with diverse plants (Brundrett 2009; Wang et al. 2017) and linked to aboveground ecosystem functions, including biomass production (Hodge and Fitter 2010; Hoeksema et al. 2010). Therefore, their presence may enhance aboveground rather than belowground ecosystem functions, suggesting that increased plant richness, through fungal composition shifts, negatively affects measured multifunctionality. Consequently, a dispersed vacant lot configuration may better support multifunctionality.

Urbanization rate indirectly affected all ecosystem functions and directly negatively impacted average multifunctionality, aligning with previous findings (Schittko et al. 2022). The multiple‐threshold approach revealed that higher multifunctionality thresholds were more susceptible to urbanization impacts, suggesting that ongoing urbanization increasingly compromises high multifunctionality levels. Urbanization‐driven biotic homogenization reduces species diversity and functional redundancy (Marcacci et al. 2021), impairing multifunctionality support (Hautier et al. 2017; Van Der Plas et al. 2016). Indeed, multifunctionality in urban parks was lower than that in natural ecosystems at the highest thresholds (Eldridge et al. 2024).

We identified the impacts of urbanization rate and vacant lot spatial configuration on ecosystem functions and multifunctionality. However, the results should be interpreted with caution because the study assessed a limited range of ecosystem functions. Urban residents depend on broader ecosystem services, including climate regulation, pollutant purification, and aesthetic value (Schwarz et al. 2017; Weiskopf et al. 2024), which were not fully captured in this analysis. As urban vacant lots become more prevalent, future research should explore additional ecosystem functions to fully elucidate their multifunctionality.

Projected population declines are anticipated to increase vacant lots. Crucially, we demonstrated that increasing urbanization and vacant lot aggregation impair multifunctionality. This suggests that assessing existing vacant lot ecosystem functions without considering their surroundings may overestimate the multifunctionality of newly emerging ones. While dispersed vacant lots in less urbanized areas can foster higher multifunctionality, this comes with a trade‐off in plant diversity. Future urban planning in shrinking cities must strategically address this conflict by integrating surrounding green space considerations and vacant lot configurations. Ultimately, maximizing vacant lot multifunctionality requires adaptive management that dynamically prioritises biodiversity conservation versus multifunctionality enhancement in changing urban landscapes.

## Author Contributions

**Yuki Iwachido:** conceptualization (equal), data curation (equal), formal analysis (lead), funding acquisition (equal), investigation (equal), methodology (equal), validation (lead), visualization (lead), writing – original draft (lead), writing – review and editing (equal). **Himari Katsuhara:** data curation (equal), formal analysis (equal), investigation (lead), writing – review and editing (equal). **Kaho Maehara:** investigation (equal), methodology (equal), writing – review and editing (equal). **Mahoro Tomitaka:** investigation (equal), writing – review and editing (equal). **Kensuke Seto:** formal analysis (equal), writing – review and editing (equal). **Shun Nonaka:** investigation (equal), writing – review and editing (equal). **Masayuki Ushio:** data curation (equal), formal analysis (equal), writing – review and editing (equal). **Maiko Kagami:** conceptualization (equal), funding acquisition (equal), project administration (equal), writing – review and editing (equal). **Takehiro Sasaki:** conceptualization (equal), funding acquisition (equal), project administration (lead), writing – review and editing (equal).

## Conflicts of Interest

The authors declare no conflicts of interest.

## Supporting information


**Data S1:** ece372995‐sup‐0001‐supinfo.pdf.

## Data Availability

The data supporting the findings of this study are openly available in Figshare at (https://doi.org/10.6084/m9.figshare.24496210.v1).
