# Supplementary material for: Urbanization and Spatial Aggregation Impair Multifunctionality in Urban Vacant Lots
Source: Ecol Evol. 2026 Jan 28;16(2):e72995. doi: 10.1002/ece3.72995 (PMC12852508; doi:10.1002/ece3.72995)
Supplement: Supplementary file 1 — Data S1: ece372995‐sup‐0001‐supinfo.pdf. [file ECE3-16-e72995-s001.pdf]

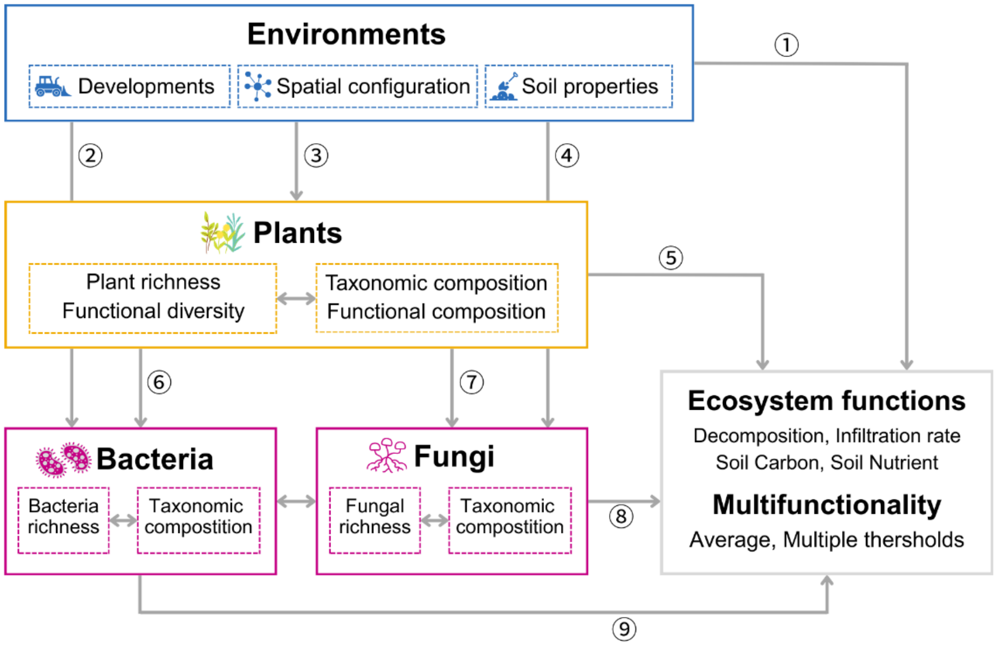

LINK	DESCRIPTION	REFERENCE
①	Several studies suggest that urbanization and soil development lead to a decline in ecosystem function and multifunctionality.	(Eldridge et al., 2024; Rivera et al., 2014; Schittko et al., 2022)
②	Land development and altered soil environmental factors (e.g., pH, nutrients, moisture) profoundly impact soil bacterial communities.	(Liu et al., 2023; Yan et al., 2016)
③	Numerous studies have demonstrated that urbanization decreases plant diversity and promotes biotic homogenization. Soil properties affect plant diversity and composition.	(Aronson et al., 2014; Koyanagi et al., 2019; Lopez et al., 2018; McKinney, 2006; Tsuzuki et al., 2020)
④	Urbanization and soil condition influence fungal richness and community composition.	(Abrego et al., 2020; Englmeier et al., 2023; Epp Schmidt et al., 2017)
⑤	Plant communities control key ecosystem functions by directly influencing nutrient cycling through uptake and litter decomposition.	(Chen et al., 2025; Tilman et al., 1997, 2014)
⑥	Plant communities shape soil bacterial assemblages through root exudates, litter inputs, and by modifying soil conditions.	(Bardgett & van der Putten, 2014; Schmid et al., 2021)
⑦	Plant communities shape soil fungal diversity and composition through litter quality, root associations (like mycorrhizae),	(Bardgett & van der Putten, 2014; Tedersoo et al., 2014)
⑧	Soil fungi drive decomposition, nutrient cycling, and plant nutrient uptake through saprotrophic and mycorrhizal activities, impacting ecosystem functions.	(Bardgett & van der Putten, 2014; Van Der Heijden et al., 2008)
⑨	Bacterial communities regulate decomposition, elemental cycling (N, C), and plant nutrient availability, impacting ecosystem functions.	(Delgado-Baquerizo et al., 2016; Wagg et al., 2019)

Fig. S1 Hypothesized structural equation modelling and a table detailing expected results, description, and references for each pathway.

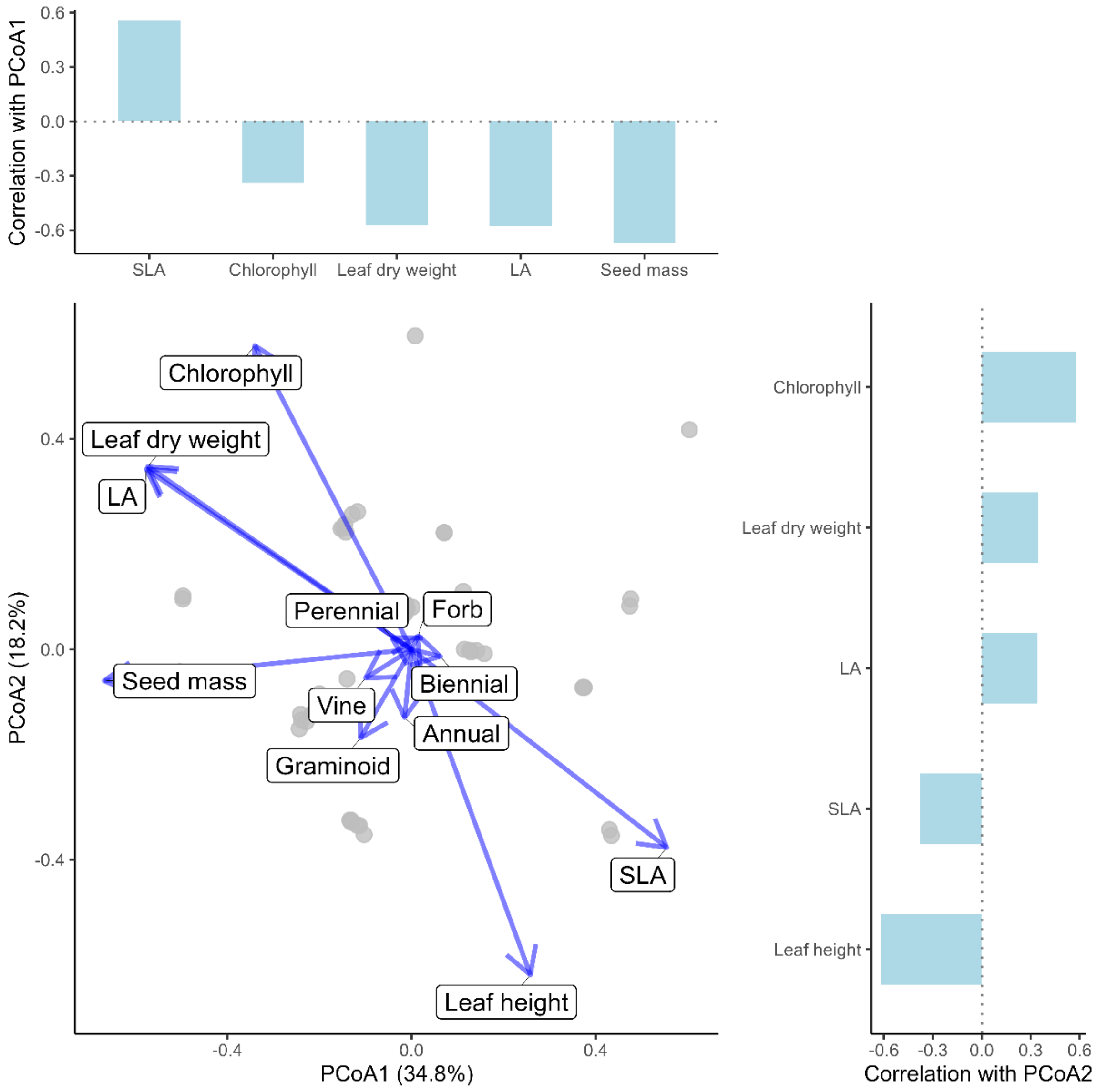

Fig S2 Principal coordinate analysis (PCoA) based on eight plant functional traits. The upper panel displays the top five correlations most strongly linked to PCoA axis 1, whereas the right-hand panel presents the top five correlations most strongly associated with PCoA axis 2.

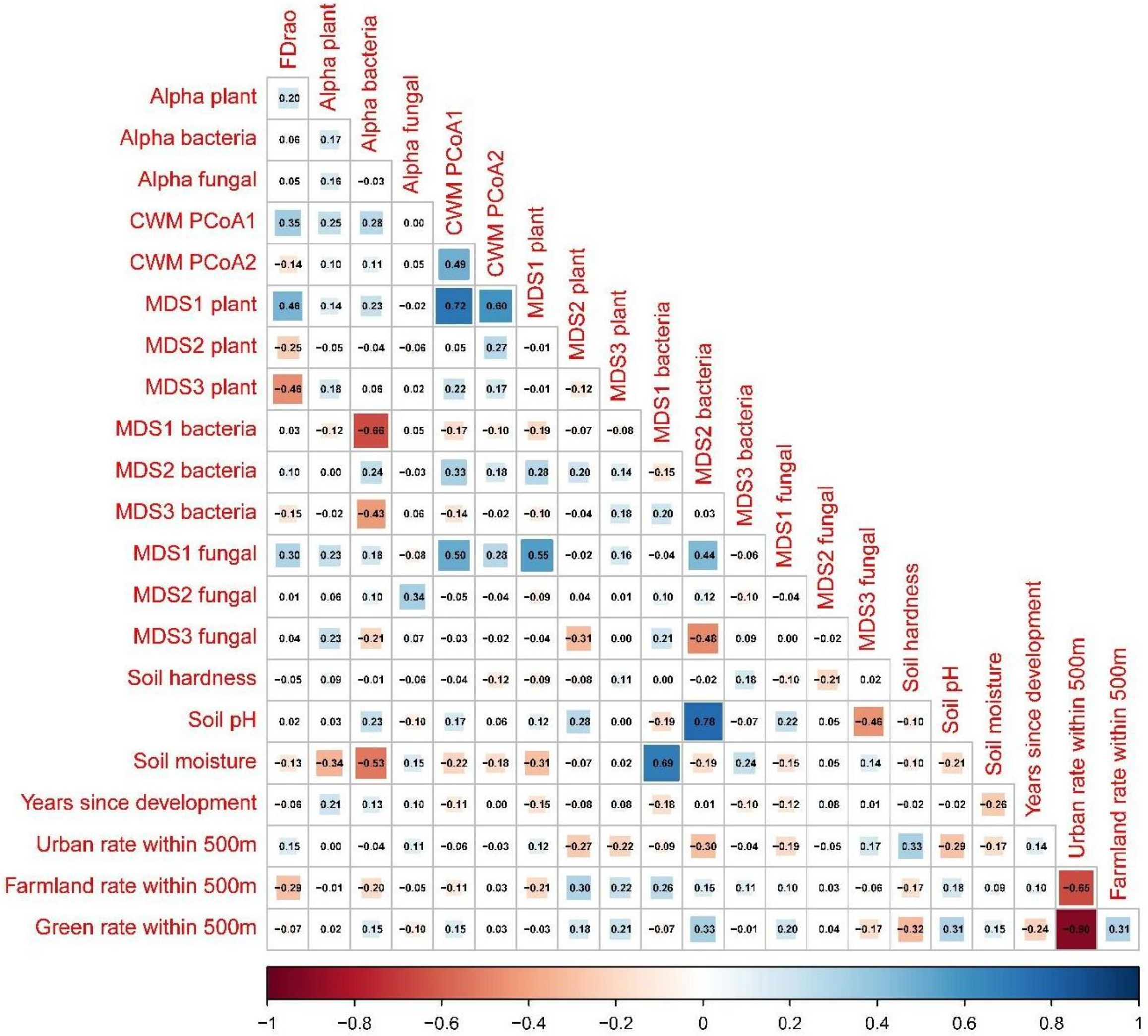

Fig. S3 Correlations between all explanatory variables.

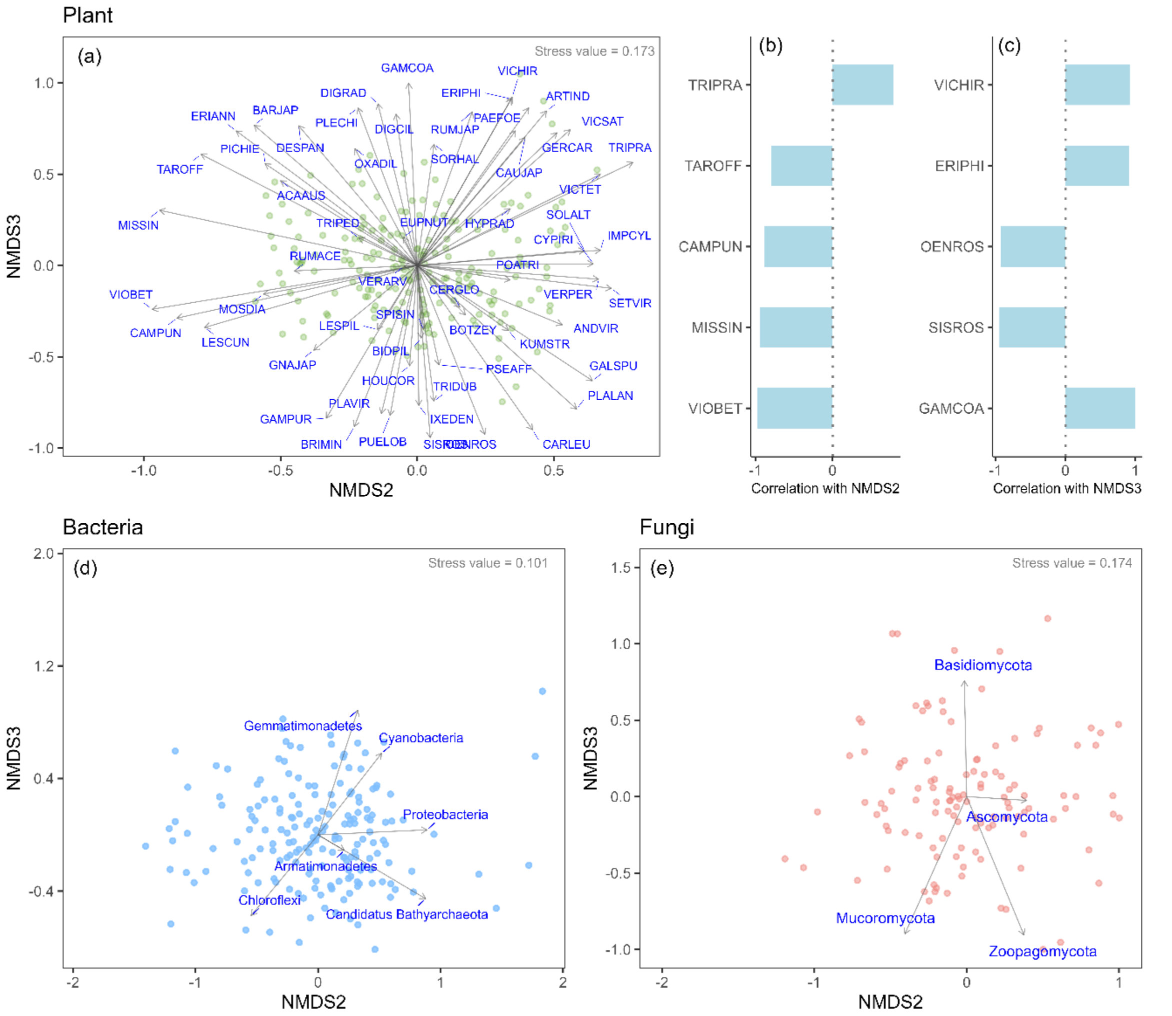

Fig. S4 Non-metric multidimensional scaling (NMDS) ordination based on Bray–Curtis dissimilarity with three dimensions for (a) plants, (d) bacteria, and (e) fungi. The upper right panels denote the top five significant correlations between plant species and NMDS (b) axis 2 and (c) axis 3. The arrows in these plots show significantly correlated NMDS axes. In plot (a), plant species abbreviation areas follows: ACAAUS (Acalypha australis), ANDVIR (Andropogon virginicus), ARTIND (Artemisia indica), BARJAP (*Barnardia japonica*), BIDPIL (*Bidens pilosa*), BOTZEY (Bothriospermum zeylanicum), BRIMIN (Briza minor), CAMPUN (Campanula punctata), CARLEU (*Carex leucochlora*), CAUJAP (*Causonis japonica*), CERGLO (*Cerastium glomeratum*), CYPIRI (*Cyperus iria*), DESPAN (*Desmodium paniculatum*), DIGCIL (*Digitaria ciliaris*), DIGRAD (*Digitaria radicosa*), ERIANN (*Erigeron annuus*), ERIPHI (*Erigeron philadelphicus*), EUPNUT (*Euphorbia nutans*), GALSPU (*Galium spurium*), GAMCOA (*Gamochaeta coarctata*), GAMPUR (*Gamochaeta purpurea*), GERCAR (*Geranium carolinianum*), GNAJAP (*Gnaphalium japonicum*), HOUCOR (*Houttuynia cordata*), HYPRAD (*Hypochaeris radicata*), IMPCYL (*Imperata cylindrica*), IXEDEN (*Ixeridium dentatum*), KUMSTR (*Kummerowia striata*), LESCUN (*Lespedeza cuneata*), LESPIL (*Lespedeza pilosa*), MISSIN (*Miscanthus sinensis*),

MOSDIA (*Mosla dianthera*), OENROS (*Oenothera rosea*), OXADIL (*Oxalis dillenii*), PAEFOE (*Paederia foetida*), PICHIE (*Picris hieracioides*), PLALAN (*Plantago lanceolata*), PLAVIR (*Plantago virginica*), PLECHI (*Pleioblastus chino*), POATRI (*Poa trivialis*), PSEAFF (*Pseudognaphalium affine*), PUELOB (*Pueraria lobata*), RUMACE (*Rumex acetosa*), RUMJAP (*Rumex japonicus*), SETFAB (*Setaria faberi*), SETVIR (*Setaria viridis*), SISROS (*Sisyrinchium rosulatum*), SOLALT (*Solidago altissima*), SORHAL (*Sorghum halepense*), SPISIN (*Spiranthes sinensis*), TAROFF (*Taraxacum officinale*), TRIDUB (*Trifolium dubium*), TRIPED (*Trigonotis peduncularis*), TRIPRA (*Trifolium pratense*), VERARV (*Veronica arvensis*), VERPER (*Veronica persica*), VICHIR (*Vicia hirsuta*), VICSAT (*Vicia sativa*), VICTET (*Vicia tetrasperma*), VIOBET (*Viola betonicifolia*).

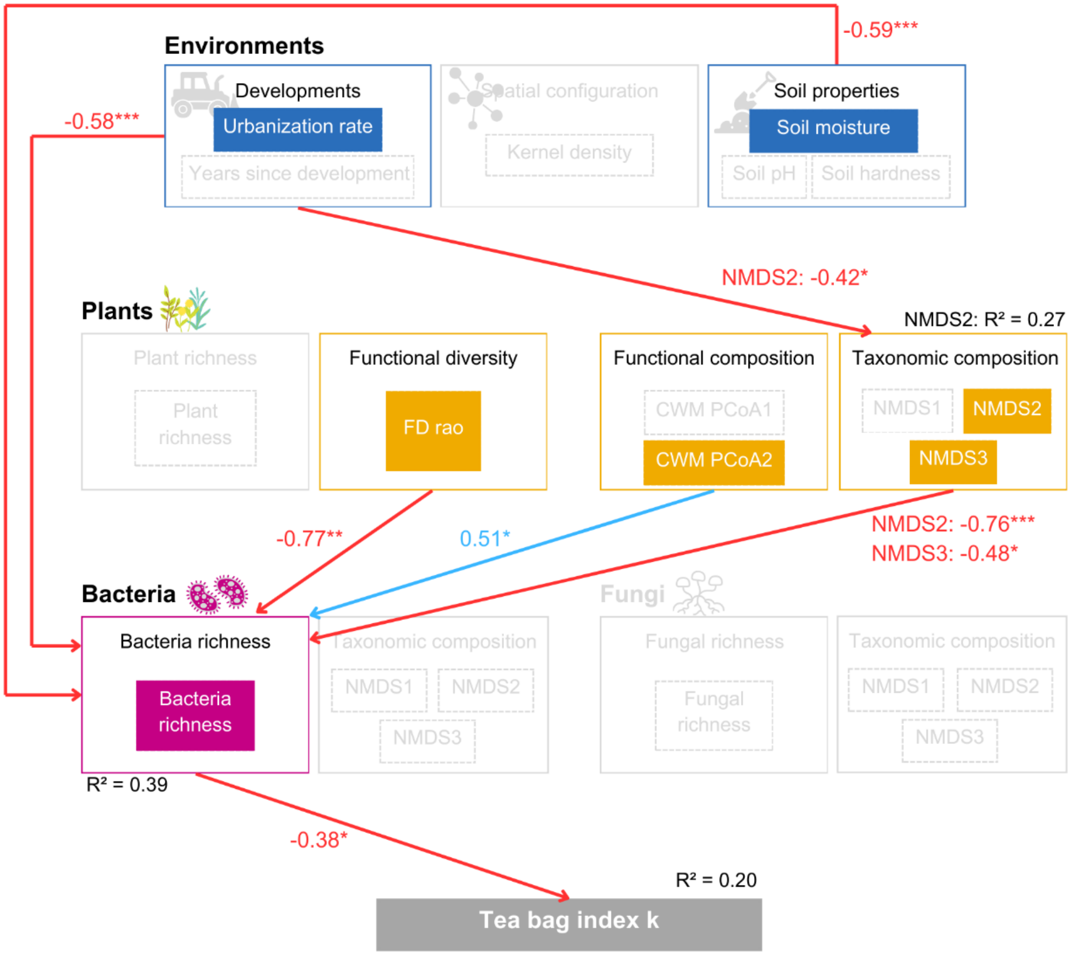

Fig. S5 Structural equation model (SEM) illustrating the direct and indirect drivers of tea bag index k (Fisher's $C = 28.786$, p-value = 0.963, AIC = -30). The blue lines indicate positive effects, while the red lines denote negative statistically significant effects. Standardized effect sizes are displayed above each path, and statistical significance is indicated by asterisks denoting p-values (* $p < 0.05$, ** $p < 0.01$, *** $p < 0.001$). The proportion of variance explained (R^2) for each variable is shown below the corresponding box. The light grey variables indicate non-significant effects on tea bag index k.

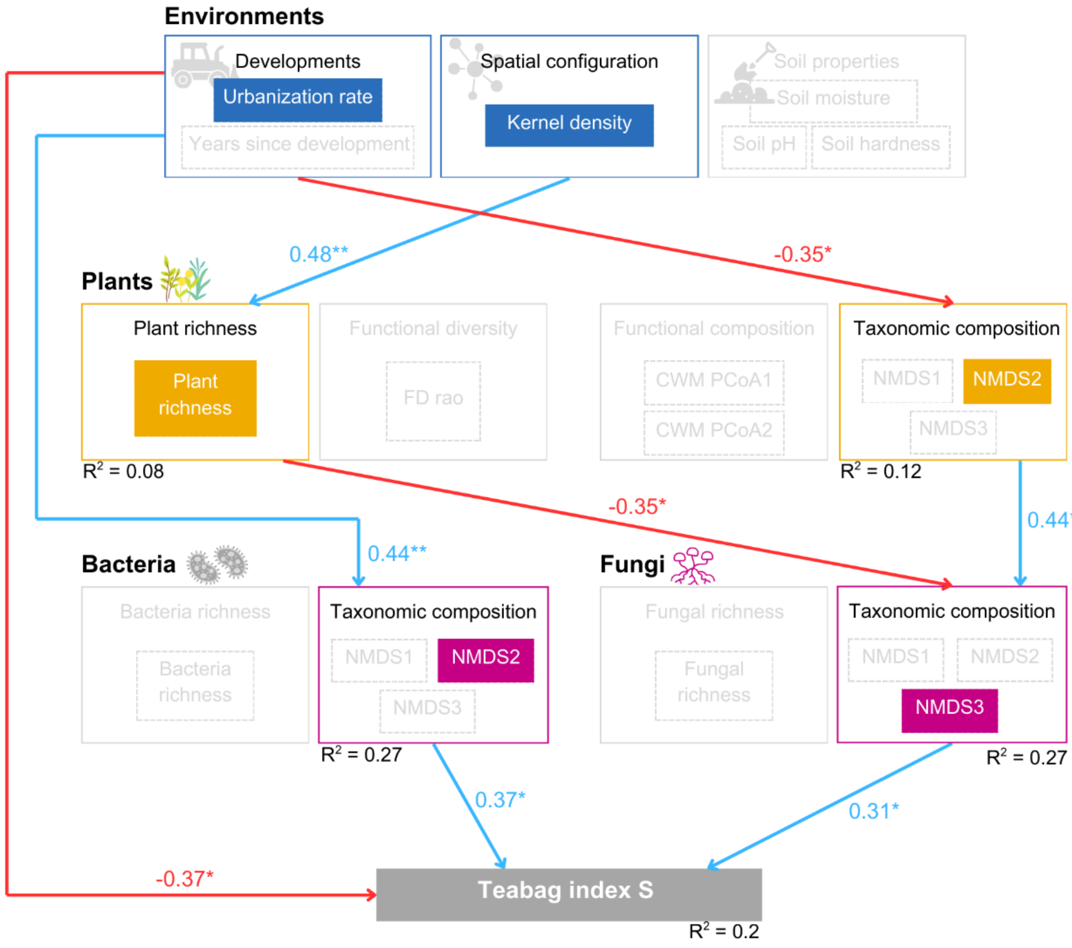

Fig. S6 Structural equation model (SEM) illustrating the direct and indirect drivers of tea bag index S (Fisher's C = 35.881, p-value = 0.901, AIC = -1.8). The blue lines indicate positive effects, while the red lines denote negative statistically significant effects. Standardized effect sizes are displayed above each path, and statistical significance is indicated by asterisks denoting p-values (* $p < 0.05$, ** $p < 0.01$, *** $p < 0.001$). The proportion of variance explained (R^2) for each variable is shown below the corresponding box. The light grey variables indicate non-significant effects on tea bag index S.

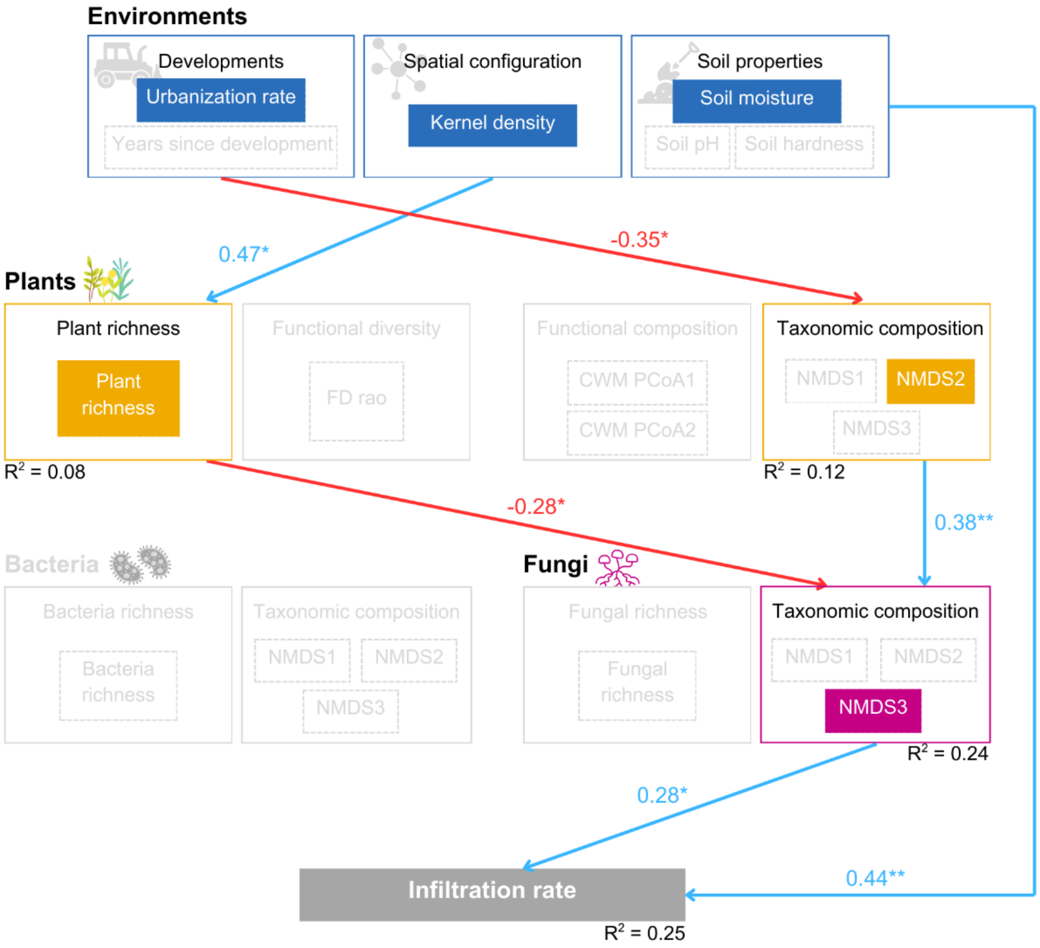

Fig. S7 Structural equation model (SEM) illustrating the direct and indirect drivers of infiltration rate (Fisher's C = 54.598, p-value = 0.376, AIC = 318). The blue lines indicate positive effects, while the red lines denote negative statistically significant effects. Standardized effect sizes are displayed above each path, and statistical significance is indicated by asterisks denoting p-values (* $p < 0.05$, ** $p < 0.01$, *** $p < 0.001$). The proportion of variance explained (R^2) for each variable is shown below the corresponding box. The light grey variables indicate non-significant effects on infiltration rate.

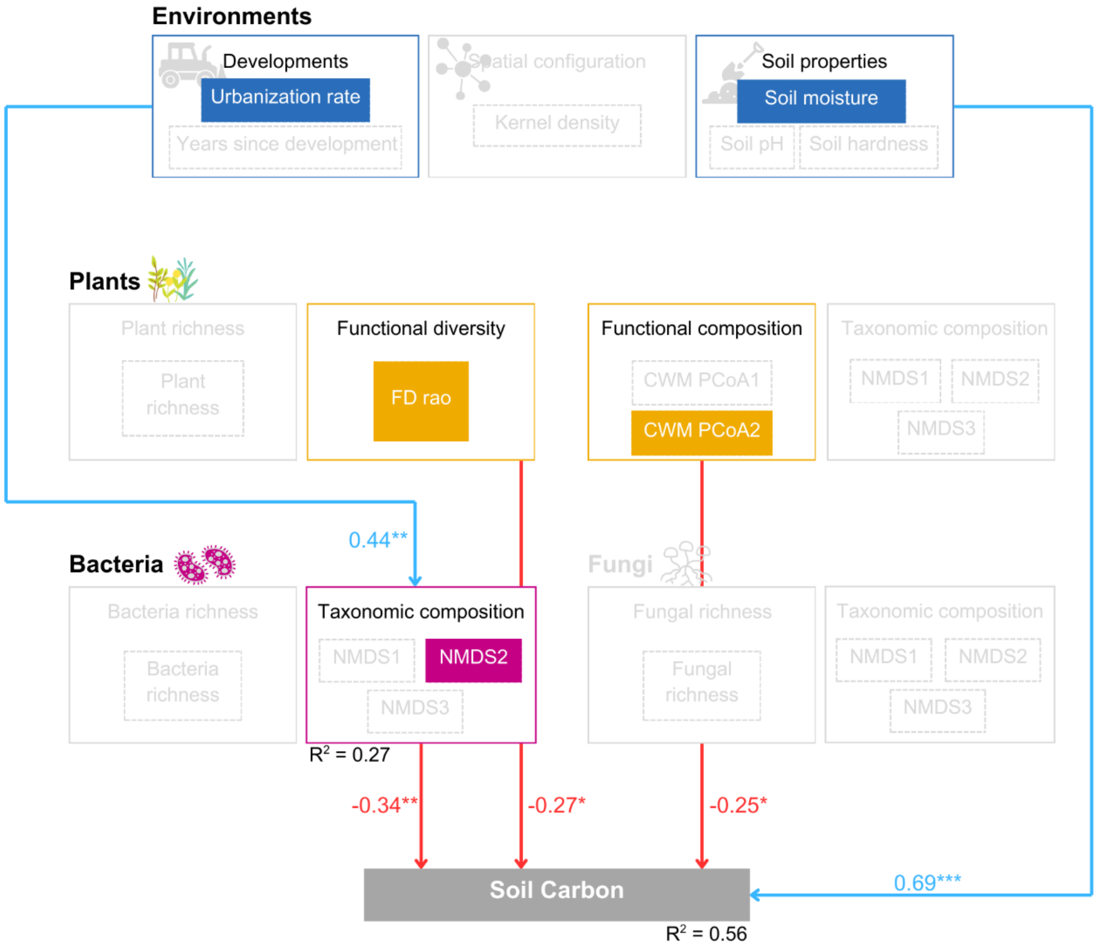

Fig. S8 Structural equation model (SEM) illustrating the direct and indirect drivers of soil carbon (Fisher's C = 27.135, p-value = 0.998, AIC = -84). The blue lines indicate positive effects, while the red lines denote negative statistically significant effects. Standardized effect sizes are displayed above each path, and statistical significance is indicated by asterisks denoting p-values (* $p < 0.05$, ** $p < 0.01$, *** $p < 0.001$). The proportion of variance explained (R^2) for each variable is shown below the corresponding box. The light grey variables indicate non-significant effects on soil carbon.

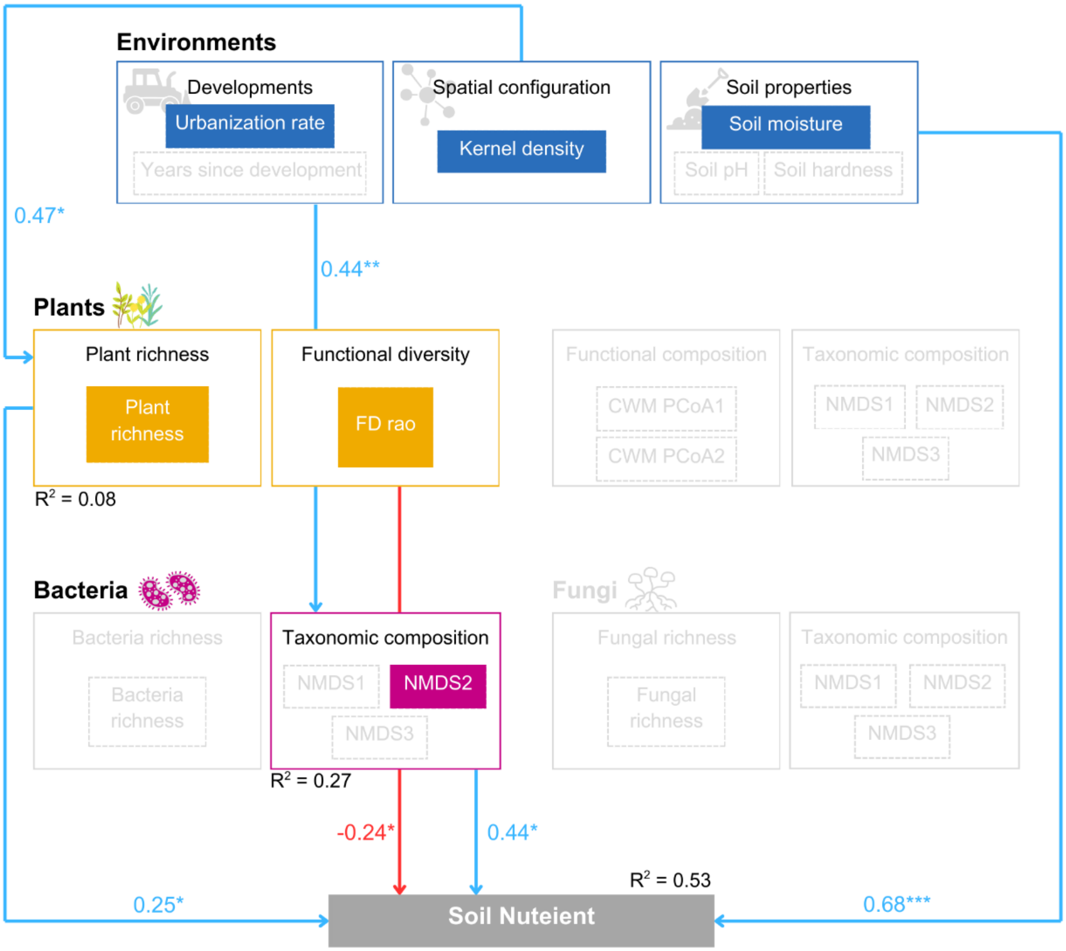

Fig. S9 Structural equation model (SEM) illustrating the direct and indirect drivers of soil nutrients (Fisher's C = 39.194, p-value = 0.935, AIC = -300). The blue lines indicate positive effects, while the red lines denote negative statistically significant effects. Standardized effect sizes are displayed above each path, and statistical significance is indicated by asterisks denoting p-values (* $p < 0.05$, ** $p < 0.01$, *** $p < 0.001$). The proportion of variance explained (R^2) for each variable is shown below the corresponding box. The light grey variables indicate non-significant effects on soil carbon.

Table S1 Summary of piecewise structural equation parsimonious models for each ecosystem function and average multifunctionality.

Response	Predictor	Estimate	Std.Error	P.Value	Std.Estimate
Teabag index k					
Teabagk	MDS2 Plant	-0.0093	0.006	0.1367	-0.3014
Teabagk	Alpha Bacteria	-1.00×10^{-4}	1.00×10^{-4}	0.0261	-0.3805
Alpha Bacteria	FDrao	-7.7898	2.6595	0.0034	-0.7685
Alpha Bacteria	CWM PCoA1	2.6785	1.1829	0.0236	0.5075
Alpha Bacteria	MDS2 Plant	-0.4585	0.1353	7.00×10^{-4}	-0.7616
Alpha Bacteria	MDS3 Plant	-0.3784	0.1901	0.0465	-0.4775
Alpha Bacteria	Soil moisture	-0.015	0.005	0.003	-0.587
Alpha Bacteria	Urbanization	-0.0108	0.0038	0.0049	-0.5811
FDrao	Urbanization	4.00×10^{-4}	4.00×10^{-4}	0.347	0.2033
CWM PCoA1	years	-9.00×10^{-4}	9.00×10^{-4}	0.3349	-0.2196
MDS2 Plant	area	-0.002	0.0012	0.1085	-0.3497
MDS2 Plant	Urbanization	-0.0128	0.006	0.0444	-0.4151
MDS3 Plant	Urbanization	-0.0065	0.0047	0.1846	-0.2776
~~FDrao	~~CWM PCoA1	0.1792	NA	0.1807	0.1792
~~FDrao	~~MDS2 Plant	-0.1285	NA	0.2574	-0.1285
~~FDrao	~~MDS3 Plant	-0.4276	NA	0.0116	-0.4276
~~CWM PCoA1	~~MDS3 Plant	0.4592	NA	0.007	0.4592
Teabag index S					
TeabagS	Alpha plant	-0.0019	0.0032	0.567	-0.0806
TeabagS	MDS3 Plant	-0.0656	0.0462	0.163	-0.1978
TeabagS	MDS3 Fungi	0.055	0.0252	0.0349	0.3051
TeabagS	MDS2 Bacteria	0.0556	0.0239	0.0251	0.3741
TeabagS	Urbanization	-0.0033	0.0015	0.0341	-0.3719
MDS3 Fungi	Alpha plant	-0.0446	0.0182	0.0193	-0.3469
MDS3 Fungi	FDrao	4.8707	4.3026	0.2637	0.1841
MDS3 Fungi	MDS2 Plant	0.6632	0.2075	0.003	0.4377
MDS3 Fungi	MDS3 Plant	0.5239	0.2872	0.0755	0.2848
MDS2 Bacteria	Alpha plant	0.0322	0.0216	0.1425	0.2066
MDS2 Bacteria	FDrao	-7.6842	4.1689	0.072	-0.2396
MDS2 Bacteria	CWM PCoA1	-3.3468	1.775	0.0663	-0.2269
MDS2 Bacteria	Urbanization	0.0261	0.009	0.007	0.4395
~~MDS3 Fungi	~~MDS2 Bacteria	-0.4898	NA	2.00×10^{-4}	-0.4898
Alpha plant	Kernel density	2.6743	1.2618	0.0341	0.4703
FDrao	Soil hardness	-0.0012	0.0024	0.6272	-0.0517
CWM PCoA1	Soil hardness	-0.0012	0.0072	0.8663	-0.0243
MDS2 Plant	Urbanization	-0.0113	0.0053	0.0414	-0.3497
MDS3 Plant	Urbanization	-0.0067	0.0045	0.1478	-0.2521
~~Alpha plant	~~CWM PCoA1	0.3692	NA	0.0045	0.3692
~~Alpha plant	~~MDS2 Plant	-0.0129	NA	0.465	-0.0129

~~Alpha plant	~~MDS3 Plant	0.1829	NA	0.1043	0.1829
~~FDrao	~~Alpha plant	0.0197	NA	0.4467	0.0197
~~FDrao	~~CWM PCoA1	-0.0893	NA	0.271	-0.0893
~~FDrao	~~MDS2 Plant	-0.054	NA	0.3563	-0.054
~~FDrao	~~MDS3 Plant	-0.4171	NA	0.0014	-0.4171
~~MDS2 Plant	~~CWM PCoA1	-0.0594	NA	0.3427	-0.0594
~~MDS3 Plant	~~CWM PCoA1	0.3836	NA	0.0033	0.3836
Infiltration rate					
Infiltration rate	MDS3 Fungi	0.9342	0.4204	0.0311	0.283
Infiltration rate	Soil moisture	0.0949	0.0281	0.0017	0.4444
MDS3 Fungi	Alpha plant	-0.0364	0.0172	0.0424	-0.2826
MDS3 Fungi	MDS2 Plant	0.5817	0.2009	0.0068	0.3839
MDS3 Fungi	MDS3 Plant	0.3408	0.24	0.1635	0.1853
Alpha plant	Kernel density	2.6743	1.2618	0.0341	0.4703
FDrao	Soil hardness	-0.0012	0.0024	0.6272	-0.0517
MDS2 Plant	Urbanization	-0.0113	0.0053	0.0414	-0.3497
MDS3 Plant	Urbanization	-0.0067	0.0045	0.1478	-0.2521
~~Alpha plant	~~MDS2 Plant	-0.0129	NA	0.465	-0.0129
~~Alpha plant	~~MDS3 Plant	0.1829	NA	0.1043	0.1829
~~FDrao	~~MDS2 Plant	-0.054	NA	0.3563	-0.054
~~FDrao	~~MDS3 Plant	-0.4171	NA	0.0014	-0.4171
Soil carbon					
Soil C	Alpha plant	0.0675	0.0383	0.087	0.1966
Soil C	FDrao	-19.0672	7.601	0.017	-0.2701
Soil C	CWM PCoA2	-8.8597	3.8208	0.0257	-0.2506
Soil C	MDS2 Bacteria	-0.7423	0.2329	0.0032	-0.3373
Soil C	Soil hardness	-0.2487	0.1639	0.1368	-0.1537
Soil C	Soil moisture	0.1199	0.0186	0	0.6941
MDS2 Bacteria	Alpha plant	0.0322	0.0216	0.1425	0.2066
MDS2 Bacteria	FDrao	-7.6842	4.1689	0.072	-0.2396
MDS2 Bacteria	CWM PCoA1	-3.3468	1.775	0.0663	-0.2269
MDS2 Bacteria	Urbanization	0.0261	0.009	0.007	0.4395
Alpha plant	Kernel density	43.7195	22.9783	0.0666	0.3194
FDrao	Urbanization	4.00×10^{-4}	3.00×10^{-4}	0.2053	0.2331
CWM PCoA1	years	-8.00×10^{-4}	9.00×10^{-4}	0.3472	-0.1632
CWM PCoA2	Soil hardness	-0.0095	0.0062	0.1323	-0.2069
~~Alpha plant	~~CWM PCoA1	0.4559	NA	5.00×10^{-4}	0.4559
~~Alpha plant	~~CWM PCoA2	0.3875	NA	0.003	0.3875
~~FDrao	~~CWM PCoA1	-0.1037	NA	0.2392	-0.1037
~~FDrao	~~CWM PCoA2	-0.2931	NA	0.0205	-0.2931
~~CWM PCoA1	~~CWM PCoA2	0.6068	NA	0	0.6068
Soil nutrient					
Soil N	Alpha plant	0.0065	0.0029	0.034	0.2522

Soil N	FDrao	-1.2389	0.5802	0.0402	-0.2352
Soil N	CWM PCoA2	-0.3376	0.2867	0.2473	-0.128
Soil N	MDS2 Bacteria	-0.0721	0.0175	3.00×10^{-4}	-0.4389
Soil N	Soil moisture	0.0088	0.0014	0	0.6803
MDS2 Bacteria	Alpha plant	0.0322	0.0216	0.1425	0.2066
MDS2 Bacteria	FDrao	-7.6842	4.1689	0.072	-0.2396
MDS2 Bacteria	CWM PCoA1	-3.3468	1.775	0.0663	-0.2269
MDS2 Bacteria	Urbanization	0.0261	0.009	0.007	0.4395
Alpha plant	Kernel density	2.6743	1.2618	0.0341	0.4703
FDrao	Urbanization	4.00×10^{-4}	3.00×10^{-4}	0.2053	0.2331
CWM PCoA1	years	-8.00×10^{-4}	9.00×10^{-4}	0.3472	-0.1632
CWM PCoA2	Soil hardness	-0.0095	0.0062	0.1323	-0.2069
~~Alpha plant	~~CWM PCoA1	0.3912	NA	0.0027	0.3912
~~Alpha plant	~~CWM PCoA2	0.2157	NA	0.0683	0.2157
~~FDrao	~~CWM PCoA1	-0.1037	NA	0.2392	-0.1037
~~FDrao	~~CWM PCoA2	-0.2931	NA	0.0205	-0.2931
~~CWM PCoA1	~~CWM PCoA2	0.6068	NA	0	0.6068
Average multifunctionality					
Average multifunctionality	MDS3 Fungi	0.0414	0.0188	0.0342	0.2123
Average multifunctionality	soil water	0.0062	0.0014	0.0001	0.49
Average multifunctionality	Urbanization	-0.0032	0.0012	0.0102	-0.3374
MDS3 Fungi	Alpha plant	-0.0364	0.0172	0.0424	-0.2826
MDS3 Fungi	MDS2 Plant	0.5817	0.2009	0.0068	0.3839
MDS3 Fungi	MDS3 Plant	0.3408	0.24	0.1635	0.1853
Alpha plant	Kernel density	2.6743	1.2618	0.0341	0.4703
MDS2 Plant	Urbanization	-0.0113	0.0053	0.0414	-0.3497
MDS3 Plant	Urbanization	-0.0067	0.0045	0.1478	-0.2521
~~Alpha plant	~~MDS2 Plant	-0.0129	-	0.465	-0.0129
~~Alpha plant	~~MDS3 Plant	0.1829	-	0.1043	0.1829
~~MDS2 Plant	~~MDS3 Plant	0.0206	-	0.4441	0.0206

Table S2 Comparison of Fisher's C and Akaike Information Criterion (AIC) values between full and optimal structural equation models (SEMs).

Ecosystem function	Model	Fisher's C	P.Value	AIC
Teabag index k	Full	63.195	< 0.001	1079.225
	Best	28.786	0.963	-30.346
Teabag index S	Full	41.38	0.028	1486.568
	Best	35.881	0.901	-14.791
Infiltration rate	Full	41.38	0.028	1658.273
	Best	54.598	0.376	318.307
Soil carbon	Full	41.38	0.028	1638.953
	Best	27.135	0.998	-94.683
Soil nutrient	Full	41.38	0.028	1487.165
	Best	39.194	0.935	-300.567
Average multifunctionality	Full	61.605	0.005	1944.375
	Best	23.347	0.613	288.243

References

Abrego, N., Crosier, B., Somervuo, P., Ivanova, N., Abrahamyan, A., Abdi, A., Hämäläinen, K., Junninen, K., Maunula, M., Purhonen, J., & Ovaskainen, O. (2020). Fungal communities decline with urbanization—More in air than in soil. The ISME Journal, 14(11), 2806–2815. https://doi.org/10.1038/s41396-020-0732-1

Aronson, M. FJ., La Sorte, F. A., Nilon, C. H., Katti, M., Goddard, M. A., Lepczyk, C. A., Warren, P. S., Williams, N. S. G., Cilliers, S., Clarkson, B., Dobbs, C., Dolan, R., Hedblom, M., Klotz, S., Kooijmans, J. L., Kühn, I., MacGregor-Fors, I., McDonnell, M., Mörtberg, U., … Winter, M. (2014). A global analysis of the impacts of urbanization on bird and plant diversity reveals key anthropogenic drivers. Proceedings of the Royal Society B: Biological Sciences, 281(1780), 20133330. https://doi.org/10.1098/rspb.2013.3330

Bardgett, R. D., & van der Putten, W. H. (2014). Belowground biodiversity and ecosystem functioning. Nature, 515(7528), 505–511. https://doi.org/10.1038/nature13855

Chen, C., Xiao, W., & Chen, H. Y. H. (2025). Meta-analysis reveals global variations in plant diversity effects on productivity. Nature, 638(8050), 435–440. https://doi.org/10.1038/s41586-024-08407-8

Delgado-Baquerizo, M., Maestre, F. T., Reich, P. B., Jeffries, T. C., Gaitan, J. J., Encinar, D., Berdugo, M., Campbell, C. D., & Singh, B. K. (2016). Microbial diversity drives multifunctionality in terrestrial ecosystems. Nature Communications, 7(1), 10541. https://doi.org/10.1038/ncomms10541

Eldridge, D. J., Cui, H., Ding, J., Berdugo, M., Sáez-Sandino, T., Duran, J., Gaitan, J., Blanco-Pastor, J. L., Rodríguez, A., Plaza, C., Alfaro, F., Teixido, A. L., Abades, S., Bamigboye, A. R., Peñaloza-Bojacá, G. F., Grebenc, T., Nahberger, T. U., Illán, J. G., Liu, Y.-R., … Delgado-Baquerizo, M. (2024). Urban greenspaces and nearby natural areas support similar levels of soil ecosystem services. Npj Urban Sustainability, 4(1), 1–9. https://doi.org/10.1038/s42949-024-00154-z

Englmeier, J., Rieker, D., Mitesser, O., Benjamin, C., Fricke, U., Ganuza, C., Haensel, M., Kellner, H., Lorz, J., Redlich, S., Riebl, R., Rojas-Botero, S., Rummler, T., Steffan-Dewenter, I., Stengel, E., Tobisch, C., Uhler, J., Uphus, L., Zhang, J., … Bässler, C. (2023). Diversity and specialization responses to climate and land use differ between deadwood fungi and bacteria. Ecography, 2023(11), e06807. https://doi.org/10.1111/ecog.06807

Epp Schmidt, D. J., Pouyat, R., Szlavecz, K., Setälä, H., Kotze, D. J., Yesilonis, I., Cilliers, S., Hornung, E., Dombos, M., & Yarwood, S. A. (2017). Urbanization erodes ectomycorrhizal fungal diversity and may cause microbial communities to converge. Nature Ecology & Evolution, 1(5), 0123. https://doi.org/10.1038/s41559-017-0123

Koyanagi, T. F., Yamada, S., Matsuzaki, H., & Kato, Y. (2019). Impacts of previous maintenance of river embankments on the grassland communities by changing soil properties. Ecological Engineering, 131, 73–80. https://doi.org/10.1016/j.ecoleng.2019.03.004

Liu, L., Zhang, Z., Wang, X., Zhang, R., Wang, M., Wurzburger, N., Li, J., & Zhang, J. (2023). Urbanization reduces soil microbial network complexity and stability in the megacity of Shanghai. Science of The Total Environment, 893, 164915. https://doi.org/10.1016/j.scitotenv.2023.164915

Lopez, B. E., Urban, D., & White, P. S. (2018). Testing the effects of four urbanization filters on forest plant taxonomic, functional, and phylogenetic diversity. Ecological Applications, 28(8), 2197–2205. https://doi.org/10.1002/eap.1812

McKinney, M. L. (2006). Urbanization as a major cause of biotic homogenization. Biological Conservation, 127(3), 247–260. https://doi.org/10.1016/j.biocon.2005.09.005

Rivera, D., Mejías, V., Jáuregui, B. M., Costa-Tenorio, M., López-Archilla, A. I., & Peco, B. (2014). Spreading Topsoil Encourages Ecological Restoration on Embankments: Soil Fertility, Microbial Activity and Vegetation Cover. PLoS ONE, 9(7), e101413. https://doi.org/10.1371/journal.pone.0101413

Schittko, C., Onandia, G., Bernard-Verdier, M., Heger, T., Jeschke, J. M., Kowarik, I., Maaß, S., & Joshi, J. (2022). Biodiversity maintains soil multifunctionality and soil organic carbon in novel urban ecosystems. Journal of Ecology, 110(4), 916–934. https://doi.org/10.1111/1365-2745.13852

Schmid, M. W., van Moorsel, S. J., Hahl, T., De Luca, E., De Deyn, G. B., Wagg, C., Niklaus, P. A., & Schmid, B. (2021). Effects of plant community history, soil legacy and plant diversity on soil microbial communities. Journal of Ecology, 109(8), 3007–3023. https://doi.org/10.1111/1365-2745.13714

Tedersoo, L., Bahram, M., Põlme, S., Kõljalg, U., Yorou, N. S., Wijesundera, R., Ruiz, L. V., Vasco-Palacios, A. M., Thu, P. Q., Suija, A., Smith, M. E., Sharp, C., Saluveer, E., Saitta, A., Rosas, M., Riit, T., Ratkowsky, D., Pritsch, K., Põldmaa, K., … Abarenkov, K. (2014). Global diversity and geography of soil fungi. Science, 346(6213), 1256688. https://doi.org/10.1126/science.1256688

Tilman, D., Isbell, F., & Cowles, J. M. (2014). Biodiversity and Ecosystem Functioning. Annual Review of Ecology, Evolution, and Systematics, 45(Volume 45, 2014), 471–493. https://doi.org/10.1146/annurev-ecolsys-120213-091917

Tilman, D., Lehman, C. L., & Thomson, K. T. (1997). Plant diversity and ecosystem productivity: Theoretical considerations. Proceedings of the National Academy of Sciences, 94(5), 1857–1861. https://doi.org/10.1073/pnas.94.5.1857

Tsuzuki, Y., Koyanagi, T. F., & Miyashita, T. (2020). Plant community assembly in suburban vacant lots depends on earthmoving legacy, habitat connectivity, and current mowing frequency. Ecology and Evolution, 10(3), 1311–1323. https://doi.org/10.1002/ece3.5985

Van Der Heijden, M. G. A., Bardgett, R. D., & Van Straalen, N. M. (2008). The unseen majority: Soil microbes as drivers of plant diversity and productivity in terrestrial ecosystems. Ecology Letters, 11(3), 296–310. https://doi.org/10.1111/j.1461-0248.2007.01139.x

Wagg, C., Schlaeppi, K., Banerjee, S., Kuramae, E. E., & van der Heijden, M. G. A. (2019). Fungal-bacterial diversity and microbiome complexity predict ecosystem functioning. Nature Communications, 10(1), 4841. https://doi.org/10.1038/s41467-019-12798-y

Yan, B., Li, J., Xiao, N., Qi, Y., Fu, G., Liu, G., & Qiao, M. (2016). Urban-development-induced Changes in the Diversity and Composition of the Soil Bacterial Community in Beijing. Scientific Reports, 6(1), 38811. https://doi.org/10.1038/srep38811
